# Capacity and Utilization of Blood Culture in Two Referral Hospitals in Indonesia and Thailand

**DOI:** 10.4269/ajtmh.17-0193

**Published:** 2017-07-10

**Authors:** Nittaya Teerawattanasook, Patricia M. Tauran, Prapit Teparrukkul, Vanaporn Wuthiekanun, David A. B. Dance, Mansyur Arif, Direk Limmathurotsakul

**Affiliations:** 1 Department of Medicine, Sunpasitthiprasong Hospital, Ubon Ratchathani, Thailand;; 2 Department of Clinical Pathology, Faculty of Medicine, Hasanuddin University/Dr. Wahidin Sudirohusodo Hospital, South Sulawesi, Indonesia;; 3 Mahidol Oxford Tropical Medicine Research Unit, Faculty of Tropical Medicine, Mahidol University, Salaya, Thailand;; 4 Lao-Oxford-Mahosot Hospital-Wellcome Trust Research Unit, Vientiane, Lao People's Democratic Republic;; 5 Centre for Tropical Medicine and Global Health, University of Oxford, Oxford, United Kingdom;; 6 Faculty of Infectious and Tropical Diseases, London School of Hygiene and Tropical Medicine, London, United Kingdom;; 7 Department of Tropical Hygiene, Faculty of Tropical Medicine, Mahidol University, Thailand

## Abstract

It is generally recommended that sepsis patients should have at least two blood cultures obtained before antimicrobial therapy. From 1995 to 2015, the number of blood cultures taken each year in a 1,100-bed public referral hospital in Ubon Ratchathani northeast Thailand rose from 5,235 to 56,719, whereas the number received in an 840-bed referral public hospital in South Sulawesi, Indonesia, in 2015 was 2,779. The proportion of patients sampled for blood cultures out of all inpatients in South Sulawesi in 2015 (9%; 2,779/30,593) was lower than that in Ubon Ratchathani in 2003 (13%; 8,707/66,515), at a time when health expenditure per capita in the two countries was comparable. Under-use of bacterial cultures may lead to an underestimate and underreporting of the incidence of antimicrobial-resistant infections. Raising capacity and utilization of clinical microbiology laboratories in developing countries, at least at sentinel hospitals, to monitor the antimicrobial resistance situation should be prioritized.

Infectious disease is still an important cause of death worldwide, and clinical microbiology laboratories play a critical role in identifying the causes of those infections. Since 2004, International Guidelines for Management of Sepsis have recommended that all patients who present with life-threatening organ dysfunction caused by a dysregulated host response to infection should have at least two blood cultures obtained before antimicrobial therapy.^1,2^ Blood cultures can be used to identify common pathogenic organisms causing both community-^3^ and hospital-acquired infections.^4^ Blood cultures are also crucial for monitoring antimicrobial resistance (AMR), both in hospitals^5^ and nationally.^6^

Although clinical microbiology laboratories are considered important, there is limited information on their capacity and use in public hospitals in developing countries.^7^ Here, we present the blood culture numbers received by a hospital microbiology laboratory in northeast Thailand, from 1995 to 2015, and compare it with the numbers received by the laboratory in a hospital in South Sulawesi, Indonesia, in 2015. The hospitals were selected due to their participation in the Southeast Asian Infectious Clinical Research Network,^8^ and their agreement to compare the activity of their clinical microbiology laboratories.

Ubon Ratchathani Province is the second largest province in northeast Thailand with a population of 1.8 million in 2015, covering 16,112 km^2^ (Figure 1). Sunpasitthiprasong Hospital is the largest hospital (1,183-bed capacity in 2015) and a referral center for all community hospitals in the province. From 1995 to 2010, Sunpasitthiprasong Hospital was the only public hospital in the province that had a clinical microbiological laboratory. In 2011 and 2015, another two community hospitals in the province (60- and 90-bed capacities) established clinical microbiological laboratories, although these tested a limited number of blood culture samples per year. South Sulawesi is a province in the southern peninsula of Sulawesi with a population of 8.4 million in 2015, and covers 46,717 km^2^. Dr. Wahidin Sudirohusodo Hospital is the largest hospital (841-bed capacity in 2015) and a referral center for all other hospitals in South Sulawesi. In 2015, there was only another single hospital in South Sulawesi, Hasanuddin University Hospital (240-bed capacity), which had a clinical microbiological laboratory. Hasanuddin University Hospital also tested a limited number of blood culture samples per year.

**Figure 1.**
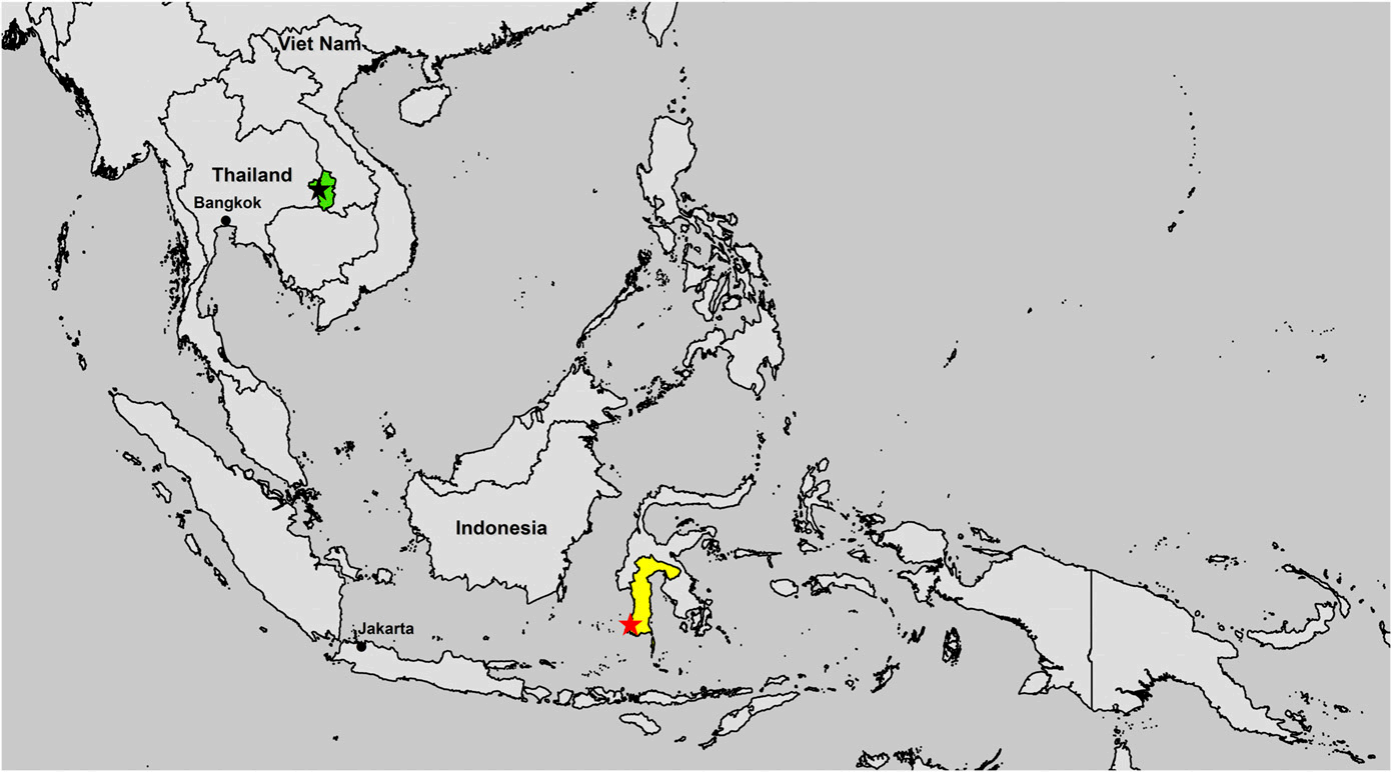
Map of Ubon Ratchathani Province (green color), location of Sunpasitthiprasong Hospital (black star), South Sulawesi Province (yellow color), and Dr. Wahidin Sudirohusodo Hospital (red star). This figure appears in color at www.ajtmh.org.

We obtained summary data based on specimens sent routinely to laboratories for clinical purposes (including the total number of blood cultures received and the total number of blood cultures positive for any pathogenic organisms) from both laboratories during the periods described earlier. As health expenditure per capita of Indonesia in 2015 was similar to health expenditure per capita in Thailand in 2003,^9^ we requested anonymized individual-level data in those years from both hospitals to estimate case-based parameters (including the total number of patients sampled for blood cultures),^6^ as this could not be estimated from the summary data. We obtained those data from the microbiology laboratory in Sunpasitthiprasong Hospital in years 2003 and 2015. We obtained only the summary data from Dr. Wahidin Sudirohusodo Hospital, and we assumed that each patient had one blood culture sample for the analysis. Because of the difficulty in establishing their clinical significance, organisms frequently associated with contamination including coagulase-negative staphylococci, viridans group streptococci, *Corynebacterium* spp. and “diphtheroids,” *Bacillus* spp., *Micrococcus* spp., *Burkholderia cepacia*, and *Propionibacterium* spp. were considered nonpathogenic.^3, 4^ The study was approved by the Institutional Review Board of Sunpasitthiprasong Hospital, and the Education and Research Department of Dr. Wahidin Sudirohusodo Hospital.

From 1995 to 2015, the number of blood cultures received each year at Sunpasitthiprasong Hospital rose from 5,235 to 56,719 (Figure 2A), whereas the number received at Dr. Wahidin Sudirohusodo Hospital in 2015 was 2,779. World bank data in 2016 showed that, from 1995 to 2015, health expenditure per capita rose from 108 to 360 current United States dollars (US$) in Thailand,^9^ whereas that in Indonesia in 2015 (99.4 current US$) was similar to that of Thailand in 2003 (Figure 2B).

**Figure 2.**
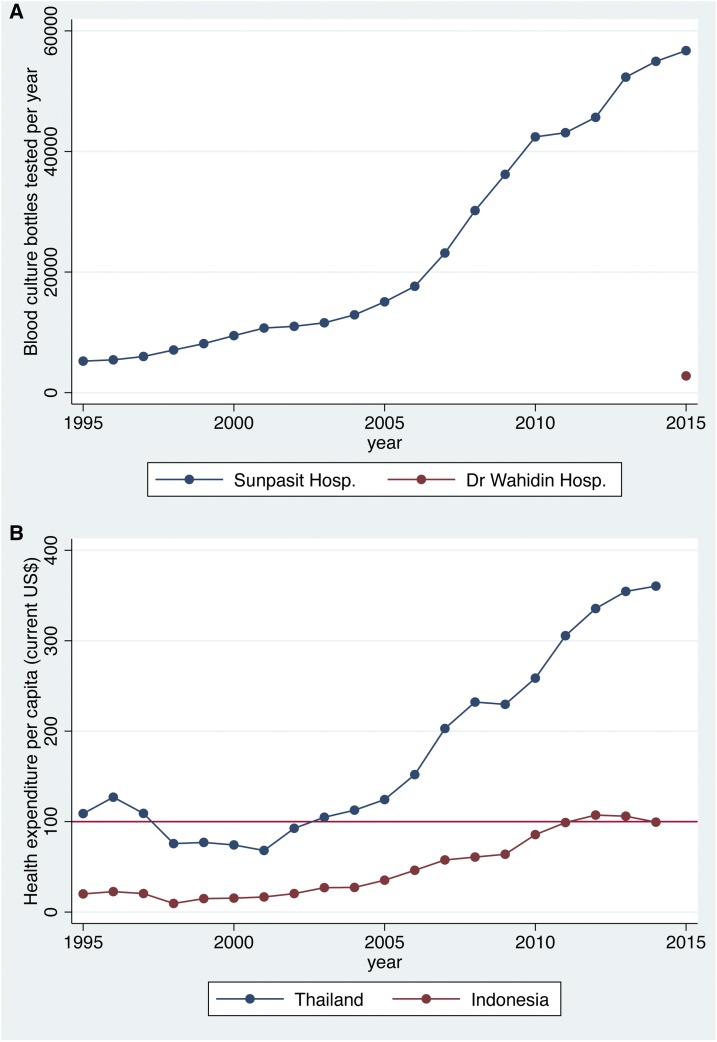
(**A**) Blood culture samples tested at Sunpasitthiprasong Hospital, Ubon Ratchathani, northeast Thailand, from 1995 to 2015 and at Dr. Wahidin Sudirohusodo Hospital, South Sulawesi, Indonesia, in 2015, and (**B**) health expenditure per capita (current US$) in Thailand and Indonesia from 1995 to 2014. This figure appears in color at www.ajtmh.org.

The Global Antimicrobial Resistance Surveillance System developed by the World Health Organization (WHO) recommends the use of the number of patients sampled for blood cultures per 100,000 inhabitants.^6^ This indicator was considerably lower at Dr. Wahidin Sudirohusodo Hospital in 2015 than that at Sunpasitthiprasong Hospital in 2003 (33 versus 485; Table 1), despite the fact that the health expenditure per capita of Indonesia in 2015 was comparable to that of Thailand in 2003. However, to take account of differing access to the health-care system and the possibility that some inhabitants may have received health care at other hospitals in the area, we also calculated the number of blood cultures taken divided by the total numbers of inpatients in the relevant year. That parameter was also lower at Dr. Wahidin Sudirohusodo Hospital in 2015 than that at Sunpasitthiprasong Hospital in 2003 (9% versus 13%; Table 1).

**Table 1:** Comparison of blood culture activity at clinical microbiology laboratories in Sunpasitthiprasong Hospital, Ubon Ratchathani, northeast Thailand, in 2003 and 2015, and in Dr. Wahidin Sudirohusodo Hospital, South Sulawesi, Indonesia, in 2015

Parameters	Sunpasitthiprasong Hospital, Thailand, in 2003	Sunpasitthiprasong Hospital, Thailand, in 2015	Dr. Wahidin Sudirohusodo Hospital, Indonesia, in 2015
General parameters for the hospitals			
Hospital bed capacity (beds)	1,099	1,183	841
Total number of inpatients (patients)	66,515	99,053	30,593
Total number of inhabitants in the catchment area	1,792,774	1,844,669	8,400,000
Capacity of clinical microbiology laboratory in the hospitals			
Number of automated blood culture machines	1 BacT/ALERT 240 (a total capacity of 240 samples)	3 BACTEC 400 (a total capacity of 1,200 samples)	2 BacT/ALERT 60 (a total capacity of 120 samples)
Number of automated microbial identification machines	None	None	Vitek 2 Compact
Number of blood cultures received	11,584	56,719	2,779
Number of patients sampled for blood cultures	8,707	20,309	2,779*
Number of patients with blood cultures positive for any pathogenic organism†	1,079	2,212	279*
Number of patients sampled for blood cultures per 100,000 inhabitants per year‡	485	1,100	33
Proportion of patients sampled for blood cultures out of all inpatients	13% (8,707/66,515)	21% (20,309/99,053)	9% (2,779/30,593)
Proportion of patients with positive blood cultures of any pathogenic organism out of all patients sampled for blood culture‡	12% (1,079/8,707)	11% (2,212/20,309)	10% (279/2,779)

* Only summary data were available at Dr. Wahidin Sudirohusodo Hospital, and we assumed that each patient had one blood culture sample in 2015.

† Coagulase-negative staphylococci, viridans group streptococci, *Corynebacterium* spp. and “diphtheroids,” *Bacillus* spp., *Micrococcus* spp., *Burkholderia cepacia*, and *Propionibacterium* spp. were considered nonpathogenic organisms.

‡ Parameters recommended by Global Antimicrobial Resistance Surveillance System developed by World Health Organization.^6^

It is worthy of note that, although the number of inpatients in Sunpasitthiprasong Hospital rose by 50% from 2003 to 2015 (from 66,515 to 99,503 patients), the number of blood cultures taken rose at a considerably higher rate (133%; from 8,707 to 20,309 sets). We also observed that in 2003, most patients (74%; 6,445/8,707) only had a single blood culture collected, whereas in 2015, most patients (80%; 16,285/20,309) had at least two blood cultures collected. This suggests that Thailand has adopted the recommendations of the “Surviving Sepsis Campaign” increasingly over time. This has not been the case in Indonesia as most patients had a single blood culture collected (Patricia M. Tauran and Mansyur Arif, personal communication). This is at least partly due to the limited resources reflected by its health expenditure in 2015 being comparable to that of Thailand in 2003.

In this study, we show that there is a clear disparity in clinical microbiological laboratory utilization between Indonesia and Thailand, even when the health expenditure per capita was taken into account. There could be multiple reasons for this. In Thailand, the cost of bacterial culture was covered by the national health-care system even before the implementation of Thailand’s Universal Healthcare Coverage Scheme in 2002.^10^ In Indonesia in 2015, the cost of bacterial culture was covered by the government only if bacterial culture was included within the package of fee for service for the final diagnosis.^11^ Thailand also has a strong infectious disease society and the importance of blood culture prior to the initiation of parenteral antimicrobials has been supported widely by the society, which probably contributes to the increased utilization of clinical microbiology laboratories in Thailand compared with that observed in Indonesia. The increase in blood culture numbers over time in Thailand was steady, and no large outbreaks of any specific bacterial infection were observed during the study period.

Although the higher proportion of patients sampled for blood cultures out of all in patients at Sunpasitthiprasong Hospital was higher than that at Dr. Wahidin Sudirohusodo Hospital (21%; 20,309/99,053 versus 9%; 2,779/30,593; Table 1), the proportions of patients with blood cultures positive for any pathogenic organism out of all patients from whom blood cultures were taken in 2015 were similar at the two hospitals (11%; 2,216/20,309 versus 10%; 279/2,779; Table 1). The difference in the proportion of patients from whom blood cultures were taken in the two hospitals is likely to indicate differences in the clinical practice of attending physicians at referral hospitals in the two countries, although it could also relate to differences in the case mix of patients presenting at each hospital. There may also have been differences in the timing of blood culture collection and pre-exposure to antibiotics. However, the fact that comparable proportions of patients with positive blood cultures were observed at both hospitals suggests that more positives would have been detected at Dr. Wahidin Sudirohusodo Hospital had more blood cultures been taken. Further studies are needed to clarify these possible differences in practice and the reasons that underlie them.

Our study has some limitations. We included data from only two public referral hospitals. Both hospitals are in southeast Asia, where sepsis and tropical infectious diseases are among the most common causes of hospital admissions.^12^ We believe that they are broadly representative of provincial hospitals in Thailand and Indonesia.

Our study supports the concerns about the available resources and underuse of microbiological laboratories in low- and middle-income countries (LMIC) raised by WHO.^13^ Both hospitals participating in our study are a part of the surveillance system for AMR in their own countries. We are also concerned that underuse of bacterial cultures may lead to an underestimate of the incidence of AMR infections (per 100,000 population for community-acquired infections^3^ and per 100,000 patient-days at risk for hospital-acquired infections^4, 5^) or of specific infections such as bacteremic melioidosis.^8^ Therefore, raising the utilization and capacity of microbiology laboratories in LMIC, at least at sentinel hospitals, to monitor the AMR situation should be prioritized.
